# Effects of *Phragmites australis* Shoot Remainder Silage on Growth Performance, Blood Biochemical Parameters, and Rumen Microbiota of Beef Cattle

**DOI:** 10.3389/fvets.2022.778654

**Published:** 2022-02-22

**Authors:** Qiye Wang, Xianglin Zeng, Yutong Zeng, Xiaoruowei Liu, Yancan Wang, Xin Wang, Jianzhong Li, Yiqiang Wang, Zhi Feng, Pengfei Huang, Jia Yin, Jing Huang, Mingzhi Zhu, Huansheng Yang

**Affiliations:** ^1^Hunan Provincial Key Laboratory of Animal Intestinal Function and Regulation, Hunan International Joint Laboratory of Animal Intestinal Ecology and Health, Laboratory of Animal Nutrition and Human Health, College of Life Sciences, Hunan Normal University, Changsha, China; ^2^Hunan Provincial Key Laboratory of Animal Nutritional Physiology and Metabolic Process, Key Laboratory of Agro-Ecological Processes in Subtropical Region, Hunan Provincial Engineering Research Center of Healthy Livestock, Scientific Observing and Experimental Station of Animal Nutrition and Feed Science in South-Central, Ministry of Agriculture, Institute of Subtropical Agriculture, Chinese Academy of Sciences, Changsha, China; ^3^Key Lab of Non-wood Forest Nurturing and Protection of National Ministry of Education, Hunan Provincial Key Laboratory for Forestry Biotechnology, Central South University of Forestry and Technology, Changsha, China; ^4^Key Laboratory of Tea Science of Ministry of Education, National Research Center of Engineering Technology for Utilization of Functional Ingredients From Botanicals, College of Horticulture, Hunan Agricultural University, Changsha, China

**Keywords:** *Phragmites australis* feed, growth performance, rumen microbiota, rumen bacterial function, beef cattle

## Abstract

The objective of the present study was to assess the effects of replacing corn silage with *Phragmites australis* shoot remainder (PSR) silage on intake, growth performance, serum biochemical parameters, and rumen microbial diversity of growing-finishing beef. Fifteen Angus beef cattle with an average body weight of 253 ± 2.94 kg were randomly divided into three groups (five replicas vs. each group vs. Angus beef cattle). The three treatments were group A fed 60% PSR silage + 40% concentrate, group B fed 30% PSR silage + 30% corn silage + 40% concentrate, and group C fed 60% corn silage + 40% concentrate. The adaptation period was 15 days, and the trial period lasted for 45 days. Results showed that the ADG was significantly higher, and FCR was significantly lower both in groups A and B compared with group C. The results of serum biochemical parameters showed that the concentration of GLU was significantly lower in group B than both groups A and C. Microbial diversity results showed that the OTUs, Shannon, Chao1, and ACE indices were significantly lower in group A compared with groups B and C. At the phyla level, the relative abundances of *Tenericutes* and *Melainabacteria* had significant differences among the three groups, and the relative abundances of *Papillibacter, Anaeroplasma*, and *Anaerovorax* had significant differences among the three groups at the genus level. Additionally, *Rikenellaceae* was the unique biomarker among the three groups. Furthermore, the results of function prediction showed that the gene families associated with metabolism of cofactors and vitamins, cellular processes and signaling, metabolism, biosynthesis of other secondary metabolites, infectious diseases, signaling molecules and interaction, nervous system, and digestive system were significantly decreased, while lipid metabolism was dramatically increased from groups A to C at KEGG level 2. At KEGG level 3, 11 metabolic pathways were significantly influenced among the three groups. In summary, these findings indicated that PSR silage substituted the corn silage totally or partially improved the growth performance, and altered the rumen microbial composition and diversity and the corresponding change in prediction function of rumen bacteria in Angus beef cattle.

## Introduction

*Phragmites australis* is a kind of native perennial grass, which is a very good non-competitive feed resource. *P. australis* has excellent nutritional value and a broad ecological distribution and adaptation in the world, and other characteristics (1). Therefore, rational utilization of *P. australis* resources is one of the effective methods to enlarge the feed source and relieve the shortage of roughage when ensuring that *P. australis* has rich nutrient content and high yield. According to statistics, China is rich in *P. australis* resources, and the distribution area is about 800,000 ha, among which Hunan has about 80,000 ha and with an annual output of up to 400,000 tons, mainly distributed in Dongting Lake and along the Yangtze River (2). *P. australis* feed has good palatability, which contains high crude protein and comprehensive mineral nutrition, and also contains a variety of amino acids and vitamins. In particular, the organic matter of starch, protein, and cellulose in *P. australis* feed degrades into monosaccharide, disaccharide, amino acid, and trace elements after fermentation, which makes the feed become soft, fragrant, and more palatable (1). Therefore, *P. australis* has the potential to be an important roughage for livestock. According to the determination (data from the American Feed Regulation Society NRC2-01-113), the dry matter of the stem and leaves of the young *P. australis* contained metabolizable energy 9.20 MJ/kg, crude protein 12.2%, crude fiber 26.8%, calcium 0.4%, and phosphorus 0.3%, which was higher than that of the common forage (3, 4). Existing studies have found that adding a certain amount of dried reed to the diet can accelerate the growth of livestock and improve the feed utilization rate (4).

Kadi et al. (5) reported that *P. australis* feed contained high N content, neutral detergent fiber (NDF), potassium, and magnesium. Tanaka et al. (1) investigated the timing of harvest and nutritive value of *P. australis* for ruminants in Lake Dianchi of China, which found that *P. australis* harvested in the early growing stage had relatively high concentrations of total digestible nutrients and demonstrated that *P. australis* can use a high-quality roughage for ruminants. Generally, there are three feed types of *P. australis* feed used in livestock: fresh, sun-dried, and ensiled. *P. australis* shoot remainder (PSR) is a by-product of processing *P. australis* shoots. By analyzing the nutrient composition of PSR, we detected that the crude protein and crude fiber contents reached 14.93 and 19.27%, respectively, which have high nutritional value, but barely have been utilized (unpublished data). While fresh PSR cannot be preserved for a long time, ensiled PSR is considered to be an effective long-term preservation method for beef cattle breeding. Different roughage may influence production performance and rumen microbial structure and function in ruminants (6–8). However, little research has indicated whether PSR can replace traditional feed ingredients in ruminants, especially affecting the rumen microbiota of beef cattle. Furthermore, effects of PSR silage on growth performance, blood biochemical indices, and rumen microbiota of beef cattle have no reports. Thus, the aim of this study was to explore the effect of PSR silage substitution for corn silage, totally or partially, on growth, serum biochemical indices, rumen microbial diversity, and predicted function in beef cattle.

## Materials and Methods

### Animals, Treatments, and Experimental Procedures

Fifteen Angus beef cattle with an average initial body weight (IBW) of 253 ± 2.94 kg were chosen and randomly allotted to three experimental treatments consisting of three dietary levels of PSR silage (DM basis): 60% (group A), 30% (group B), and 0 (group C) as a substitute of corn silage, respectively. Experimental diets were composed of 60% of silage and 40% of concentrate (DM basis) and were formulated to meet nutritional requirements (9) of beef cattle, and feed ingredients, and the nutritional composition are shown in Table 1. Each bull was fed in individual pens with automatic drinking and free feeding intake, five pens per group. Before the trial, all bulls were weighed, dewormed, and vaccinated (foot and mouth disease vaccine and anthrax vaccine). Cattle were adapted to the diets for 15 days, and the experimental period lasted for 45 days.

**Table 1:** Diet ingredients and nutrition levels.

**Item**	**Groups**		
	**A**	**B**	**C**
**Ingredient, %**			
PSR silage	60	30	0
Corn silage	0	30	60
Corn	16.00	16.00	16.00
Wheat bran	14.30	14.30	14.30
Soybean meal	7.20	7.20	7.20
NaCl	0.50	0.50	0.50
Premix^a^	2.00	2.00	2.00
Total	100	100	100
**Nutrient levels^b^**			
NE_mf_, MJ /kg	7.99	8.04	8.07
Crude protein, %	14.39	13.30	12.22
Crude fat, %	2.89	2.84	2.79
Neutral-detergent fiber, %	33.85	34.76	35.66
Acid-detergent fiber, %	23.56	25.40	27.24
Ca, %	0.37	0.36	0.35
P, %	0.30	0.29	0.27

a *Premix provides the following per kg: vitamin A 160,000 IU, vitamin D3 22,000 IU, vitamin E 1,200 mg, Cu 380 mg, Fe 1,100 mg, Zn 1,900 mg, Mn 1,600 mg, I 20 mg, Se 5.8 mg, Co 2.5 mg*.

b *Except for NE_mf_, which was the predicted value referring to the related formulas of Feeding Standard of Beef Cattle (NY/T 815-2004), the rest was measured value*.

Bulls were fed three times daily at 07:00, 12:00, and 17:00 h with total mixed diets. Residual feed was evaluated at 06:00 h each day to quantify and adjust daily feed allowance to a maximum of 5% residues. Feed samples were collected from each pen every 15 days and then composited, and were frozen at −20°C for nutritional ingredients analysis.

### Growth Performance and Blood Biochemical Parameters

Each bull was weighed individually in the morning on an empty stomach at the beginning and end of the experiment. The ADG (average daily gain) was calculated by the weight gain per bull divided by the trial days. The ADFI (average daily feed intake) was calculated by the amount of diet offered minus the residues per pen and then divided by the total trial days. The FCR (feed conversion ratio) was calculated as ADFI per ADG (10). At the end of the experiment, blood samples were collected in a 5-ml vacuum tube without anticoagulant (Changsha Yiqun Medical Equipment) from the caudal vein of each bull in the morning on an empty stomach. After standing for 2–3 h, blood samples were centrifuged by the model TG16-WS H1650 centrifuges (Hunan Xiangyi Laboratory Instrument Development Co. Ltd.) at 3,000 r/min for 10 min, and then the supernatant was separated and stored at −20°C for further analysis. A TBA-120FR automatic biochemistry analyzer (Toshiba Corporation) was used to measure the concentrations of serum biochemical parameters (11–13).

### DNA Extraction and Amplification of 16S rRNA Genes

The total microbial genomic DNA was extracted using the CTAB/SDS method. The V3–V4 regions of 16S rRNA genes were amplified with forward primer V515F (5′-GTGYCAGCMGCCGCGGTAA-3′) and reverse primer V806R (5′-GGACTACHVGGGTWTCTAAT-3′). The PCR reactions were performed in 30-μl systems. For specific PCR amplified procedures, refer to Wang et al. (14, 15). The sequencing libraries were constructed by Ion Plus Fragment Library Kit 48 rxns (Thermo Scientific). The Ion S5^TM^ XL platform to sequence was further used, and 407- to 412-bp single-end reads were generated (14, 15).

### Sequencing and Bioinformatics Analysis

The raw reads were cleaned by the Cutadapt quality control process (16). The UCHIME algorithm (17) was used to detect and remove the chimera sequences and finally to obtain the clean reads. Sequence analysis was performed by Uparse software (Uparse v7.0.1001) (18) to cluster the operational taxonomic units (OTUs) with ≥97% similarity. The Silva Database (19) was used to annotate taxonomic information and normalize the OTU abundant information. The alpha diversity and beta diversity were analyzed subsequently by QIIME (Version 1.7.0) and displayed by R Software (Version 2.15.3). Phylogenetic Investigation of Communities by Reconstruction of Unobserved States (PICRUSt) was utilized to predict the metabolic function of the microbiota. The raw sequencing data of this study were submitted to the Sequence Read Archive (SRA) with accession numbers SRR15662882–SRR15662896.

### Statistical Analysis

The experimental data were analyzed on SPSS 22.0 software packages (SPSS, Chicago, IL, USA). Using the one-way ANOVA and *t*-tests to test the significance of growth performances and serum biochemical parameters, and the non-parameter test was performed to analyze the rumen microbial diversities, relative abundance, and function prediction. Final results were presented with meaning values. Differences were considered to have a tendency at 0.05 < *p* < 0.10 and statistically significant at *p* ≤ 0.05.

## Results

### Growth Performance

The results of growth performance are shown in Table 2. There was no significant difference in IBW (initial body weight) and ADFI among treatments, although the FBW (final body weight) did not differ among treatments and was higher in groups A and B than in group C. The ADG was significantly greater (linear, *p* = 0.032) in groups A and B than in group C. Similarly, the FCR was significantly lower (linear, *p* = 0.006) in groups A and B than in group C. Notably, the ADG and FCR were not different between A and B treatments.

**Table 2:** Effects of PSR silage on growth performance of Angus beef cattle (*N* = 5).

**Item^2^**	**Groups^1^**			**SEM^3^**	***p*** **-value**	
	**A**	**B**	**C**		**Linear**	**Quadratic**	
Initial weight, kg	250.00	252.00	257.00	8.78	0.767	0.942
Final weight, kg	313.00	315.00	304.00	9.10	0.719	0.764
ADG, kg/day	1.40^a^	1.40^a^	1.04^b^	0.07	0.032	0.186
ADFI, kg/day	6.58	7.11	6.77	0.29	0.800	0.517
FCR	4.71^b^	5.09^b^	6.63^a^	0.33	0.006	0.269

*Values within a row with different superscripts (a, b) differ significantly at p < 0.05*.

1 *A, fed 60% PSR silage; B, fed 30% PSR silage + 30% corn silage; C, fed 60% corn silage*.

2 *ADG, average daily gain; ADMI, average daily feed intake; FCR, feed conversion rate*.

3 *SEM, standard error of the mean; PSR, phragmites australis shoots remainder*.

### Serum Biochemical Parameters

The concentrations of serum TP, ALT, AST, TG, CHOL, HDL, LDLC, and NH3 did not differ among treatments. The concentration of serum GLU was quadratically affected (quadratic, *p* = 0.004) among treatments, and the concentration of BUN showed a linearly downward trend (linear, *p* = 0.096) among treatments (Table 3).

**Table 3:** Effects of PSR silage on serum biochemical parameters of Angus beef cattle (*N* = 5).

**Item^2^**	**Groups^1^**			**SEM^3^**	***p*** **-value**	
	**A**	**B**	**C**		**Linear**	**Quadratic**
TP, g/L	70.28	69.10	73.02	1.05	0.300	0.267
GLU, mmol/L	3.76^a^	3.14^b^	4.06^a^	0.14	0.246	0.004
BUN, mmol/L	6.04	5.82	5.18	0.20	0.096	0.619
ALT, U/L	31.28	30.94	29.38	1.36	0.604	0.847
AST, U/L	82.40	82.40	67.20	5.19	0.256	0.505
TG, mmol/L	0.24	0.26	0.30	0.02	0.239	0.776
CHOL, mmol/L	2.26	2.46	2.15	0.11	0.672	0.297
HDL, mmol/L	2.11	2.25	1.93	0.10	0.486	0.313
HDLC, mmol/L	0.65	0.72	0.54	0.04	0.344	0.200
NH3, mmol/L	177.70	162.04	162.24	4.67	0.193	0.430

*Values within a row with different superscripts (a, b) differ significantly at p < 0.05*.

1 *A, fed 60% PSR silage; B, fed 30% PSR silage + 30% corn silage; C, fed 60% corn silage*.

2 *TP, total protein; GLU, glucose; BUN, blood urea nitrogen; ALT, alanine aminotransferase; AST, aspartate aminotransferase; TG, triglyceride; CHOL, cholesterol; HDL, high-density lipoprotein; LDLC, low-density lipoproteincholesterol; NH3, ammonia*.

3 *SEM, standard error of the mean*.

### Rumen Bacterial Communities

**Figure 1:**
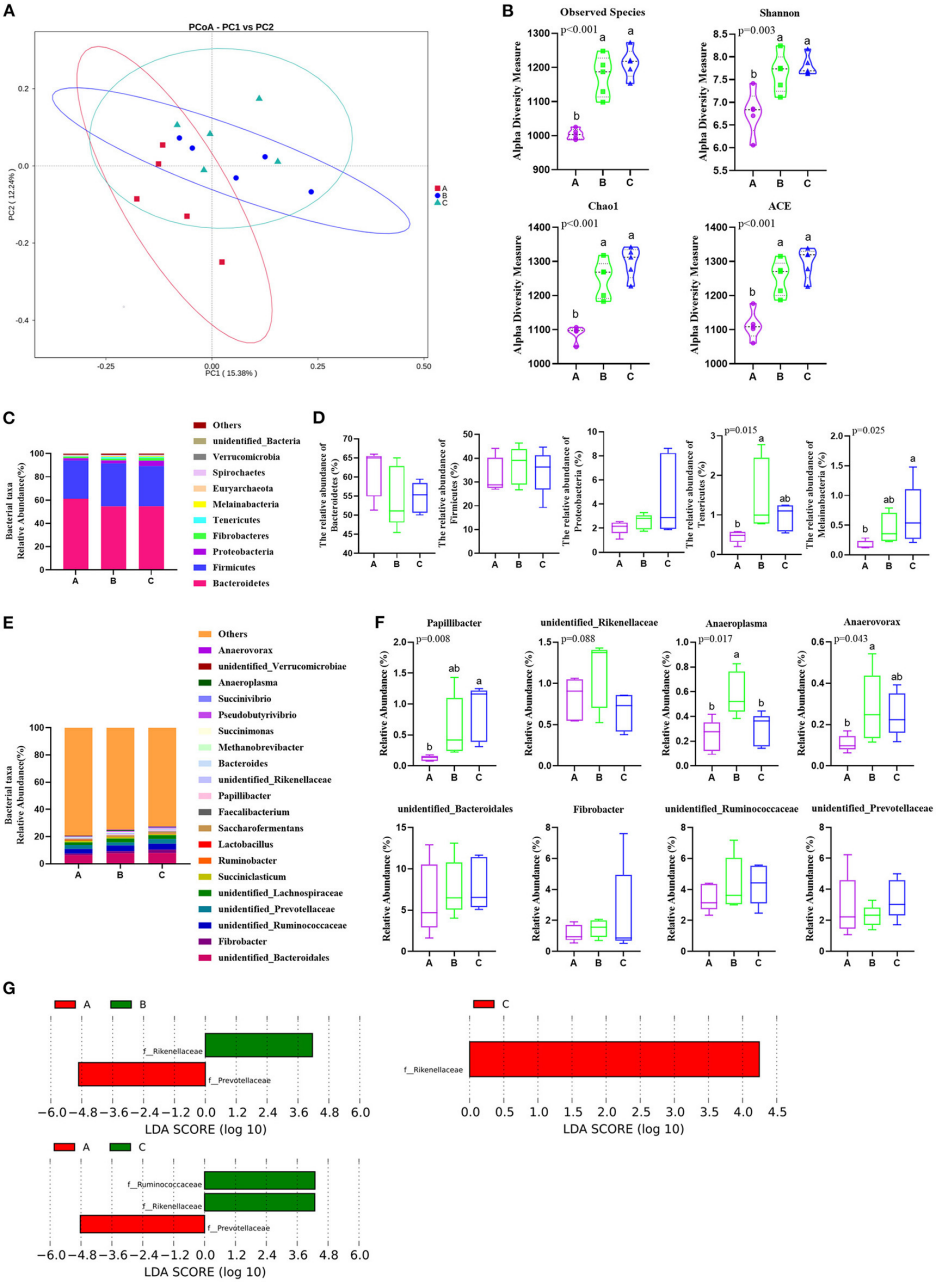
*Phragmites australis* shoot remainder (PSR) silage totally or partially substituting the corn silage alters rumen microbiota composition in Angus beef cattle (*N* = 5). Group A, fed 60% PSR silage; Group B, fed 30% PSR silage + 30% corn silage; and Group C, fed 60% corn silage. **(A)** PCoA analysis of rumen microbiota based on operational taxonomic unit (OTU) abundance. **(B)** Assessment of alpha diversity. **(C)** Rumen microbiota taxonomic profiling at the phylum level. **(D)** Relative abundances of Bacteroidetes, Firmicutes, Proteobacteria, Tenericutes, and Melainabacteria. Bars with different letters (a, b) indicate significant differences (*p* < 0.05) among different groups (the same below). **(E)** Rumen microbiota taxonomic profiling at the genus level. **(F)** Relative abundances of representative and significant difference genera. **(G)** LDA score of rumen microbiota composition according to LEfSe analysis by three treatments.

The results of sequencing analysis are presented in Figure 1. The difference in bacterial composition among the three groups was analyzed by the PCoA, and the PCoA plots showed that the group A data had a tendency to be separated from both the B and C groups (Figure 1A). Similarly, there were no significant differences in diversity and uniformity by the level of species richness existing between B and C based on the observed species (OTUs), Shannon, Chao1, and Ace index analyses (Figure 1B). Compared with both groups B and C, the A group had less OTUs (*p* < 0.001), Chao1 (*p* < 0.001), ACE (*p* < 0.001), and Shannon (*p* < 0.01) unexpectedly (Figure 1B).

A total of 22 bacterial phyla were identified by taxonomic analysis in the rumen samples. The relative abundance of more than 1% were *Bacteroidetes, Firmicutes, Proteobacteria*, and *Fibrobacteres* (Figures 1C,D). Notably, the relative abundance of *Bacteroidetes* and *Firmicutes* was the richest in the three trial groups (Figures 1C,D). Additionally, the relative abundance of *Tenericutes* (*p* = 0.015) and Melainabacteria (*p* = 0.025) was significantly lower in group A than that in group B and group C, respectively, at the phyla level (Figure 1D).

**Figure 2:**
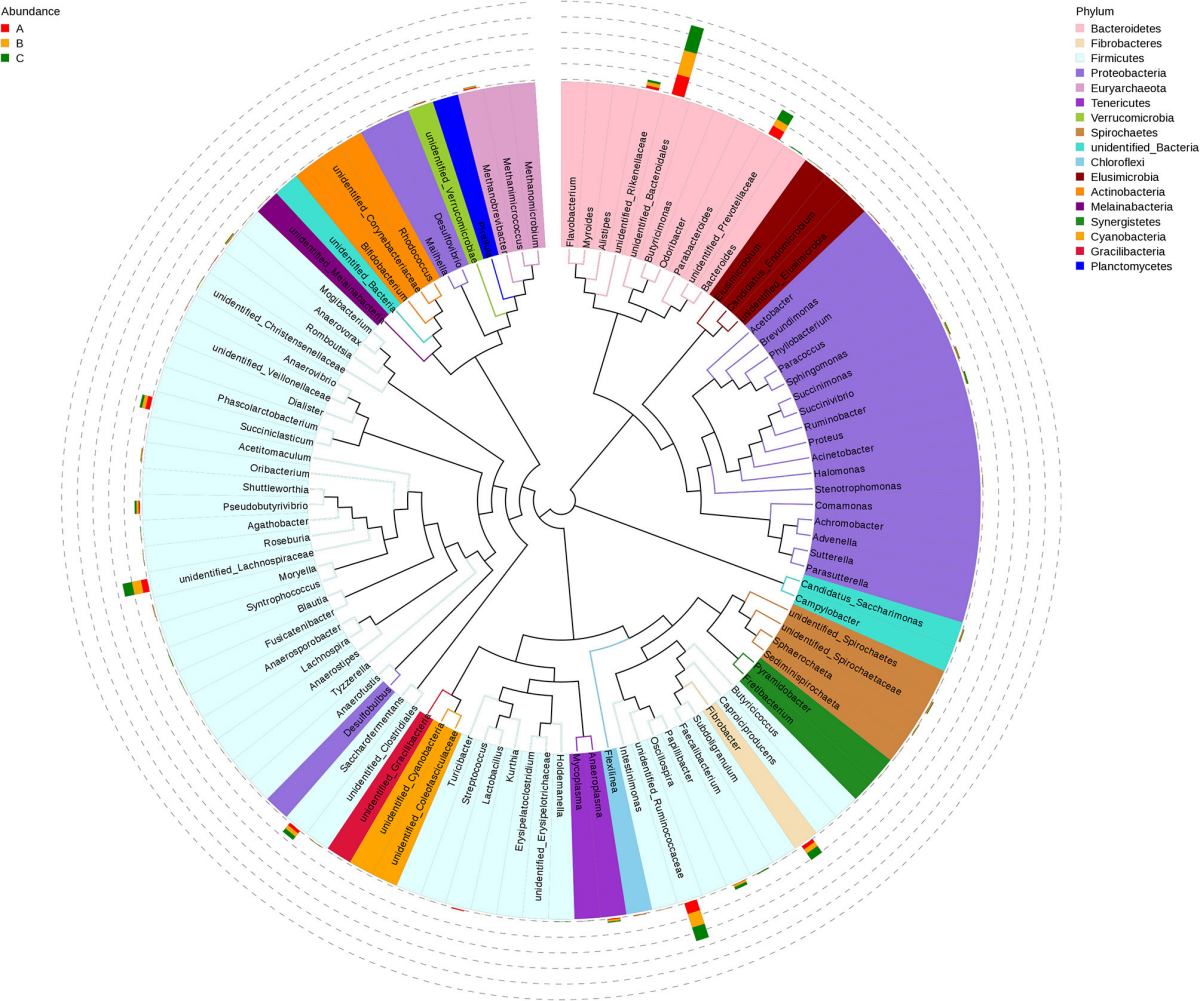
Phylogenetic tree of genus level species among PSR silage totally or partially substituted the corn silage groups in Angus beef cattle (*N* = 5). Group A, fed 60% PSR silage; Group B, fed 30% PSR silage + 30% corn silage; and Group C, fed 60% corn silage. Branches and fan colors represent its corresponding phyla, the accumulation histogram outside the fan ring shows the relative abundance distribution information of the genus in different groups.

A total of 122 bacterial genera were detected at the genus level. Twenty representative genera were elucidated in all the rumen samples (Figure 1E). Among these genera, the relatively high abundance (>1%) *unidentified_Bacteroidales* belonged to *Bacteroidetes* in the phylum, *Fibrobacter* is one of *Fibrobacteres, unidentified_Ruminococcaceae*, and *unidentified_Lachnospiraceae* belong to *Clostridia* in members of *Firmicutes*. *Unidentified_Prevotellaceae* belongs to *Bacteroidetes, Succiniclasticum* is also a member of *Firmicutes*. The relative abundances of *Papillibacter* (*p* = 0.008), *Anaeroplasma* (*p* = 0.017), and *Anaerovorax* (*p* = 0.043) had significant differences among the three groups, and the relative abundance of *unidentified_Rikenellaceae* (*p* = 0.088) also had a notable change in group B in the genus level (Figure 1F). Additionally, LEfSe analysis results showed the dominant bacteria species for each group by LDA score, the *Prevotellaceae* and *Rikenellaceae* showed statistical differences and were considered as biomarkers between groups A and B, the *Prevotellaceae, Ruminococcaceae*, and *Rikenellaceae* were the biomarkers between groups A and C, and the *Rikenellaceae* was the unique biomarker among the three groups (Figure 1G). Furthermore, the phylogenetic tree of the top 100 genera was obtained through multisequence alignment, as shown in Figure 2, in which the phylogenetic relationship of rumen bacteria species at the genus level could be presented more intuitively among the three groups.

### Predicted Metabolic Pathways and Functions of Rumen Bacterial Communities

**Figure 3:**
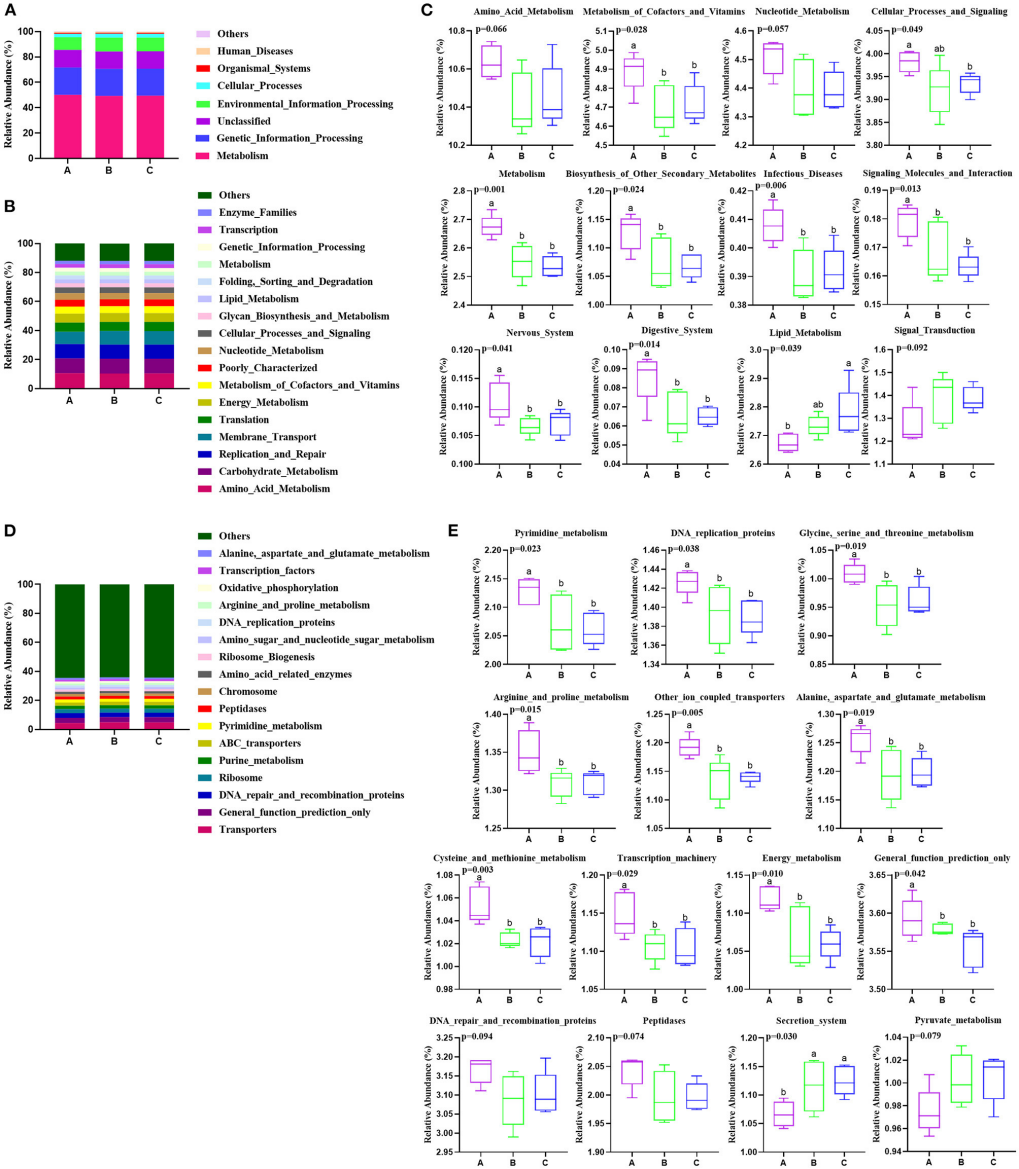
Effects of PSR silage totally or partially substituted the corn silage on the predicted functional composition of rumen bacterial community in Angus beef cattle (*N* = 5). Group A, fed 60% PSR silage; Group B, fed 30% PSR silage 30% corn silage; and Group C, fed 60% corn silage. **(A)** The majority of the gene sequences annotated to KEGG level 1. **(B)** The majority of the gene sequences annotated to KEGG level 2. **(C)** The gene families of significant differences at KEGG level 2. **(D)** The majority of the gene sequences annotated to KEGG level 3. **(E)** The relative abundant pathways with significant differences at KEGG level 3. Bars with different letters (a,b) indicate significant differences (*p* < 0.05) among different groups.

Metabolic functions of rumen bacteria were predicted by PICRUSt in the present study (Figure 2). The result showed that “metabolism” was in the highest relative abundance with more than 49% of all sequence reads among three groups at KEGG level 1 (Figure 3A). At KEGG level 2, the most relatively abundant gene families (relative abundance > 0.10%) from all rumen samples are present in Figure 3B. Genes belonging to amino acid metabolism, carbohydrate metabolism, replication and repair, membrane transport, translation, and energy metabolism were the most relative abundant among the three groups (Figure 3B). Among these gene families, the genes associated with metabolism of cofactors and vitamins (*p* = 0.028), cellular processes and signaling (*p* = 0.049), metabolism (*p* = 0.001), biosynthesis of other secondary metabolites (*p* = 0.024), infectious diseases (*p* = 0.006), signaling molecules and interaction (*p* = 0.013), nervous system (*p* = 0.041), and digestive system (*p* = 0.014) were significantly decreased from groups A to C, amino acid metabolism (*p* = 0.066) and nucleotide metabolism (*p* = 0.057) showed the descent tendency, while lipid metabolism (*p* = 0.039) was dramatically increased, and signal transduction had an increasing tendency (*p* = 0.092) from groups A to C (Figure 3C). At KEGG level 3, the majority of relatively abundant pathways were transporters, general function prediction only, DNA repair and recombination proteins, ribosome, purine metabolism, and ABC transporters (Figure 3D). Notably, the relative abundance of 11 pathways showed significant variation among the three groups (Figure 3E). The pathways involved in the pyrimidine metabolism (*p* = 0.023), DNA replication proteins (*p* = 0.038), glycine, serine, and threonine metabolism (*p* = 0.019), arginine and proline metabolism (*p* = 0.015), other ion coupled transporters (*p* = 0.005), alanine, aspartate, and glutamate metabolism (*p* = 0.019), cysteine and methionine metabolism (*p* = 0.003), transcription machinery (*p* = 0.029), energy metabolism (*p* = 0.010), and general function prediction only (*p* = 0.042) were significantly increased, and DNA repair and recombination proteins (*p* = 0.094) and peptidases (*p* = 0.074) had increased trend in group A compared with the other two groups. Inversely, secretion system (*p* = 0.030) was significantly decreased, and pyruvate metabolism (*p* = 0.079) had a decreased trend in group A than in the other two groups B and C.

## Discussion

Depending on the results of animal feeding trial, PSR silage group (group A), mixed group (group B), and corn silage (group C) affected the growth performance of Angus beef cattle. The ADG was significantly affected by PSR silage, which in PSR silage group was the highest, followed by the mixed group, equally 34.62% higher than corn silage group. Inversely, the FCR in the PSR silage group and mixed group was obviously lower than in the corn silage, 31.52 and 23.23% lower, respectively, while the ADFI had no significant difference among the three groups. These results indicated that PSR silage substitution for corn silage totally or partially could improve the growth performance of beef cattle, mainly by improving feed utilization efficiency to increase the ADG in the growing–fattening of Angus beef cattle. Thus, it can be observed that PSR silage might completely replace corn silage for beef cattle breeding. Therefore, the effective use of *P. australis* and other unconventional feed materials may not only expand feed sources and reduce feeding costs but also further improve the weight gain rate and meat production performance of beef cattle, and finally increase the economic benefits of breeding.

Nutrients are digested and absorbed by the body and carried through the bloodstream to tissues, organs, and cells. Therefore, blood biochemical indicators can be a good response to the intake of nutrition levels of the body. GLU concentration can reflect the energy metabolism of animals (20). In the current study, the concentration of GLU in the mixed group was significantly lower than in the PSR silage and corn silage treatments, while the higher ADG was observed in the mixed group. These results were inconsistent with previous studies, and this might be related to species and feed composition (11–15). The concentration of BUN is perceived as an effective indicator to measure the metabolism of protein and amino acid, low BUN level indicates high nitrogen metabolism capacity (21). Diets supplemented with PSR silage increased the concentrations of BUN in the present study, which might be related to the high protein content of *P. australis* shoots remainder.

Rumen is the most powerful digestive organ, in which complex microbial communities are closely related to degrade and convert plant materials in ruminants (22). More than 70% of the energy was provided by rumen bacteria fermentation to ensure the host growth and reproduction performance (23), and the compositions of rumen microorganism are influenced by diets (24). In the present study, the core microbiome accounts for more than 78% of total OTUs among the three groups, and this result was similar with other researches (11–13). The core microbiome plays crucial roles in maintaining the “functional redundancy” of rumen ecosystem, and this redundancy further guarantees its major functional properties (25). The present study showed that the observed species (OTUs), Shannon, Chao1, and ACE indices were significantly decreased in the PSR silage diet, indicating that the rumen microbiome was altered by PSR silage.

The present study systematically revealed the composition and structure of rumen microbiome in Angus beef cattle fed PSR silage. Bacteroidetes and Firmicutes were the most two relatively abundant phyla in the current study, which was referred to the degradation of protein and carbohydrates (26), and these findings were consistent with previous studies (27). de Menezes et al. (28) have also found that the dairy cows fed pasture or TMR diets did not have obvious differences on the rumen microbiome at the phylum level, with the sequences of *Bacteroidetes* and *Firmicutes* representing approximately 80% of the total rumen microbiome. Consistently, the relative abundance of *Bacteroidetes* and *Firmicutes* were over 80% in all three diets in the current study. Proteobacteria was detected as the third most relatively abundant phylum in this study, which was similar to previous studies (29). In general, the composition and structure of rumen microbial community might be related to the feed efficiency and animal breed. Studies have found that several members belonged to the phylum *Tenericutes* related to being animal pathogens and parasites, and the greater abundance of *Tenericutes* was associated with the reduced intramuscular fat deposition of longissimus in Angus steers (30, 31). The relative abundance of *Tenericutes* was the lowest in the PSR silage diet in the present study, indicating that PSR silage might improve gastrointestinal health and further promotes intramuscular fat deposition. Actually, the growth performance was the greatest by feeding PSR silage diet. *Melainabacteria* is a newly identified gut bacteria, whose relative abundance in the rectal contents of diabetic model rats was significantly increased (32). The relative abundance of *Melainabacteria* was significantly decreased (especially in group A) after adding PSR silage to the diets in the present study, which indicated that PSR silage-substituted corn silage might improve host sugar, fat, and protein metabolism.

At the genus level, the effects of PSR silage on the rumen bacterial population were further identified in this study. The relative abundance of *Papillibacter* in the PSR silage group was significantly lower than that in the corn silage and mixed groups. *Papillibacter* is known as a butyrate producer (33). The decreased abundance of *Papillibacter* in PSR silage indicated that the butyrate production was relatively decreased by rumen microbiota. The relative abundance of *Anaeroplasma* in the mixed group was significantly higher than the groups of corn silage and PSR silage. *Anaeroplasma* is a genus characterized by its anaerobic fermentation, which produces fatty acids as propionate (34). All of these taxa were previously reported as members of the regular and efficient microbiota from rumen, and their increased abundance may indicate an improved ability of digestion or, at least, a need for more specialized fermentation in rumen due to, for example, more food intake. Besides, *Anaeroplasma* was highly correlated with high weight gain, and may be important for cattle nutrition either individually or in a consortium (35).

Some studies indicate that *Anaerovorax* can generate more SCFAs to provide additional energy sources and maintain feed efficiency (36). Unexpectedly, the relative abundance of *Anaerovorax* in the PSR silage group is significantly lower than that in the corn silage and mixed groups in the present study. The relative abundance of *unidentified_Rikenellaceae* in the corn silage group was obviously lower than that in the PSR silage and mixed groups. To date, all cultured members of the family *Rikenellaceae* are described as anaerobic, mesophilic, and rod-shaped bacteria that usually ferment carbohydrates or proteins. Su et al. (37) isolated a carbohydrate-fermenting and hydrogen-producing *Rikenellaceae* from a reed swamp in China (37). It remains to identify and examine the functions of the *unidentified Rikenellaceae* to understand the roles in the present study. Additionally, the PSR silage diet increased the relative proportion of *Rikenellaceae* at the family level, and *Rikenellaceae* might be one of the biomarkers between PSR silage feed and corn silage feed using LEfSe.

The composition of rumen microorganisms affects host metabolic function and physiological health. The relative abundance of the dominant microbial phyla is stable in ruminants. The dominant three microbial phyla were *Bacteroidetes, Firmicutes*, and *Proteobacteria* in the rumen among the three groups, indicating that the rumen microbiota in cattle was also relatively stable at the phyla level. These results were in agreement with previous studies (38, 39), and the most dominant phyla *Firmicutes* and *Bacteroidetes* were closely related to carbohydrate and protein metabolism (40, 41). At the genus level, the dominant four genera were *unidentified Bacteroidales, Fibrobacter, unidentified Ruminococcaceae*, and *unidentified Prevotellaceae*, and their relative abundance all did not have significant difference among different diets in the present study, which was similar to previous studies (41, 42). Menni et al. (43) found that the abundance of *Ruminococcaceae* might play a significant role in energy and lipid metabolism, and which was negatively associated with vascular sclerosis. These results indicated that the PSR silage diet might not affect host health in cattle.

The PICRUSt prediction results showed that amino acid metabolism, carbohydrate metabolism, replication and repair, membrane transport, translation, and energy metabolism were the dominant gene families at KEGG level 2, all of which are essential for survival, growth, and reproduction of gastrointestinal microbes (44). These results were similar to our previous studies in sheep (11–15). Among these gene families, unexpectedly, the genes associated with metabolism of cofactors and vitamins, cellular processes and signaling, metabolism, biosynthesis of other secondary metabolites, infectious diseases, signaling molecules and interaction, nervous system, and digestive system were significantly higher in the PSR silage diet than in the mixed and corn silage diets, while lipid metabolism was dramatically lower in the PSR silage diet than in the corn silage diet. Furthermore, the majority of gene families were transporters, general function prediction only, DNA repair, and recombination proteins, ribosome, purine metabolism, and ABC transporters at KEGG level 3. Notably, the relative abundance of 11 pathways showed significant variation among the three groups. The pathways involved in the pyrimidine metabolism, DNA replication proteins, glycine, serine, and threonine metabolism, arginine and proline metabolism, other ion coupled transporters, alanine, aspartate, and glutamate metabolism, cysteine and methionine metabolism, transcription machinery, energy metabolism, and general function prediction only were significantly higher, and secretion system was significantly lower in the PSR silage diet than in the mixed and corn silage diets. These results indicated an enhanced fermentation activity performed by rumen microorganisms in the PSR silage diet. The current study also implied that feeding only a roughage of PSR silage diet altered the ruminal microbial functions.

In conclusion, the present study mainly investigated that the growth performance, blood biochemical parameters, and the composition and function of rumen microbiota of PSR silage feed totally or partially substituted the corn silage in Angus beef. The results suggest that the PSR silage diet and mixed diet increase ADG and decrease FCR, reduce serum glucose levels, and alter the rumen microbiota and inferred metabolic functions. These findings indicated that PSR silage could partially substitute corn silage for beef cattle breeding, replacing 30% of corn silage in the diet has good feeding effect in cattle.

## Conclusions

Feeding different PSR silage level diets improved growth performance, changed the contents of serum glucose and urea nitrogen and, furthermore, might affect the energy and protein metabolic efficiency of Angus beef. Moreover, the rumen bacterial diversity indices decreased significantly by feeding PSR silage, the relative abundances of *Tenericutes* and *Melainabacteria* were significantly reduced by feeding PSR silage, and *Papillibacter, Anaeroplasma*, and *Anaerovorax* had significantly decreased by feeding PSR silage at the genus level, and furthermore, the metabolic pathways were significantly influenced by related bacteria for PSR silage. The results indicated that feeding PSR silage could improve the growth performance and alter the rumen bacteria diversity and the corresponding function, and PSR silage could partially substitute (30%) corn silage for beef cattle breeding.

## Data Availability Statement

The datasets presented in this study can be found in online repositories. The names of the repository/repositories and accession number(s) can be found below: https://www.ncbi.nlm.nih.gov/, SRR15662882-SRR15662896.

## Ethics Statement

The experimental procedures of this study were approved by the Animal Care Committee of Hunan Normal University in reference to the Administration of Affairs Concerning Experimental Animals.

## Author Contributions

HY and QW conceptualized the study. XZ and XL handled the methodology. PH and ZF were in charge of the software. MZ was responsible for the validation. YZ and YaW performed the formal analysis. QW conducted the investigation, prepared and wrote the original draft, and handled the project administration. HY handled the resources and acquired the funding. XW and JH were responsible for the data curation. MC and YiW assisted in the writing, review, and editing of the draft. MZ and JY handled the visualization. JL was in charge of the supervision. All authors have read and agreed to the published version of the manuscript.

## Funding

This work was supported by the Hunan Provincial Key Laboratory of Animal Nutritional Physiology and Metabolic Process open fund projects (ISA2020113), Scientific Research Project of Hunan Education Department (No. 20B369), Young Elite Scientists Sponsorship Program by CAST (YESS20160086), and Hunan Province's Strategic and Emerging Industrial Projects (2018GK4035).

## Conflict of Interest

The authors declare that the research was conducted in the absence of any commercial or financial relationships that could be construed as a potential conflict of interest.

## Publisher's Note

All claims expressed in this article are solely those of the authors and do not necessarily represent those of their affiliated organizations, or those of the publisher, the editors and the reviewers. Any product that may be evaluated in this article, or claim that may be made by its manufacturer, is not guaranteed or endorsed by the publisher.
